# Impact of Probiotic *B. infantis* EVC001 Feeding in Premature Infants on the Gut Microbiome, Nosocomially Acquired Antibiotic Resistance, and Enteric Inflammation

**DOI:** 10.3389/fped.2021.618009

**Published:** 2021-02-16

**Authors:** Marielle Nguyen, Heaven Holdbrooks, Prasanthi Mishra, Maria A. Abrantes, Sherri Eskew, Mariajamiela Garma, Cyr-Geraurd Oca, Carrie McGuckin, Cynthia B. Hein, Ryan D. Mitchell, Sufyan Kazi, Stephanie Chew, Giorgio Casaburi, Heather K. Brown, Steven A. Frese, Bethany M. Henrick

**Affiliations:** ^1^Neonatology, Kaiser Permanente Orange County, Anaheim, CA, United States; ^2^Evolve Biosystems Inc., Davis, CA, United States; ^3^Department of Food Science and Technology, University of Nebraska Lincoln, Lincoln, NE, United States; ^4^Department of Nutrition, University of Nevada, Reno, NV, United States

**Keywords:** preterm infant, gut microbiome, enteric inflammation, antibiotic resistant genes (ARG), *Bifidobacterium longum* subspecies *infantis* EVC001

## Abstract

**Background:** Preterm birth is a major determinant of neonatal survival and morbidity, but the gut microbiome and associated enteric inflammation are also key factors in neonatal development and the risk of associated morbidities. We prospectively and longitudinally followed two cohorts of preterm infants, one of which was fed activated *Bifidobacterium longum* subsp. *infantis* (*B. infantis*) EVC001 8 × 10^9^ CFU daily, and the other was not fed a probiotic. Hospital feeding protocol assigned all infants born at <1500 g and/or < 32 weeks corrected gestational age to the probiotic feeding protocol, whereas infants born at >1500 g and/or >32 weeks corrected gestational age were not fed a probiotic. Fecal samples were opportunistically collected from 77 infants throughout the hospital stay, and subjected to shotgun metagenomic sequencing and quantification of enteric inflammation. De-identified metadata was collected from patient medical records.

**Results:** The gut microbiome of preterm infants was typified by a high abundance of *Enterobacteriaceae* and/or *Staphylococcaceae*, and multivariate modeling identified the probiotic intervention, rather than degree of prematurity, day of life, or other clinical interventions, as the primary source of change in the gut microbiome. Among infants fed *B. infantis* EVC001, a high abundance of total *Bifidobacteriaceae* developed rapidly, the majority of which was *B. infantis* confirmed via subspecies-specific qPCR. Associated with this higher abundance of *Bifidobacteriaceae*, we found increased functional capacity for utilization of human milk oligosaccharides (HMOs), as well as reduced abundance of antibiotic resistance genes (ARGs) and the taxa that harbored them. Importantly, we found that infants fed *B. infantis* EVC001 exhibited diminished enteric inflammation, even when other clinical variables were accounted for using multivariate modeling.

**Conclusion:** These results provide an important observational background for probiotic use in a NICU setting, and describe the clinical, physiological, and microbiome-associated improvements in preterm infants associated with *B. infantis* EVC001 feeding.

## Introduction

Preterm birth, defined as <37 weeks gestation age, accounts for over 1 in 10 live births in the United States and is associated with an immature gastrointestinal tract, diminished gut barrier function, and underdeveloped immune function leading to increased morbidity and mortality compared to term infants (1–3). Unlike infants born at full-term, premature infants have extended stays in the hospital environment of the Neonatal Intensive Care Unit (NICU), increased exposure to associated clinical protocols including antibiotics, proton pump inhibitors, parenteral nutrition, and limited access to human milk, all of which dramatically shapes gut microbiome composition (4, 5). Importantly, recent work has shown that preterm infants are rapidly colonized by nosocomial, antibiotic-resistant bacteria associated with an increased risk of serious infection and death (6). These bacteria confer negative impacts on neonatal growth and development (4) and are associated with an increased risk of necrotizing enterocolitis (NEC) (7, 8) and late-onset sepsis (9–11). Importantly, increased prevalence of *Proteobacteria* has been shown to precede NEC (7, 11, 12) and exacerbate enteric inflammation (13). *Proteobacteria*, broadly, have also been associated with the pathogenesis of sepsis and NEC in premature infants (14–16). Although the exact mechanisms by which these bacteria evoke heightened inflammatory responses in the preterm infant gut are not completely understood, suboptimal mucosal integrity and/or predisposition toward an exaggerated inflammatory profile, including exacerbated IL-8 cytokine production, has been observed in preterm infants (17). Indeed, exaggerated inflammatory responses driven by nosocomially derived microbes including *Proteobacteria* have led to a desire to find strategies, including the use of probiotics, to mitigate the risks associated with prematurity that have etiological links to the gut microbiome.

A recent observational study comparing longitudinal fecal samples taken from preterm infants showed that supplementation with *Bifidobacterium* and *Lactobacillus* remodeled the gut microbiome, replicating a gut microbiome more closely resembling that of a term infant. However, physiological effects on the host were not determined (18), and distinct species-specific effects among *Bifidobacterium* are beginning to emerge that distinguish the ability among *Bifidobacterium* species to modulate host enteric inflammation and epithelial integrity from those that do not (19, 20). Beyond observational studies, extensive evidence has indicated a beneficial role of feeding *Bifidobacterium longum* subsp. *infantis* (*B*. *infantis*) to premature infants, with significant decreases in morbidity and mortality reported, including those from NEC (21–23). In animal models, *B. infantis* supplementation was effective at reducing NEC injury scores and minimizing intestinal inflammation (24, 25). Furthermore, establishing *B. infantis* abundance in the infant gut restores important ecosystem services of the microbiome that are beneficial to infant health (26). Particularly, the efficient fermentation of human milk oligosaccharides (HMOs) into host-accessible organic acids (e.g., acetate and lactate) helps reduce degradation of gastrointestinal mucin, and results in significant reductions of bacterial populations with pathogenic potential (19, 26–29). Moreover, when *B. infantis* is compared directly to other probiotic bacteria, including *Bifidobacterium animalis* subsp. *lactis* (*B. lactis*), it showed superior ability to colonize the premature infant gut (30), based on its ability to utilize the full suite of selective prebiotic carbohydrates found in human milk (HMOs). Therefore, early establishment of a gut microbiome that limits growth of bacteria associated with detrimental outcomes, maximizes nutrition, and reduces inflammation during a key developmental window is particularly important to preterm neonatal health.

**Figure 1:**
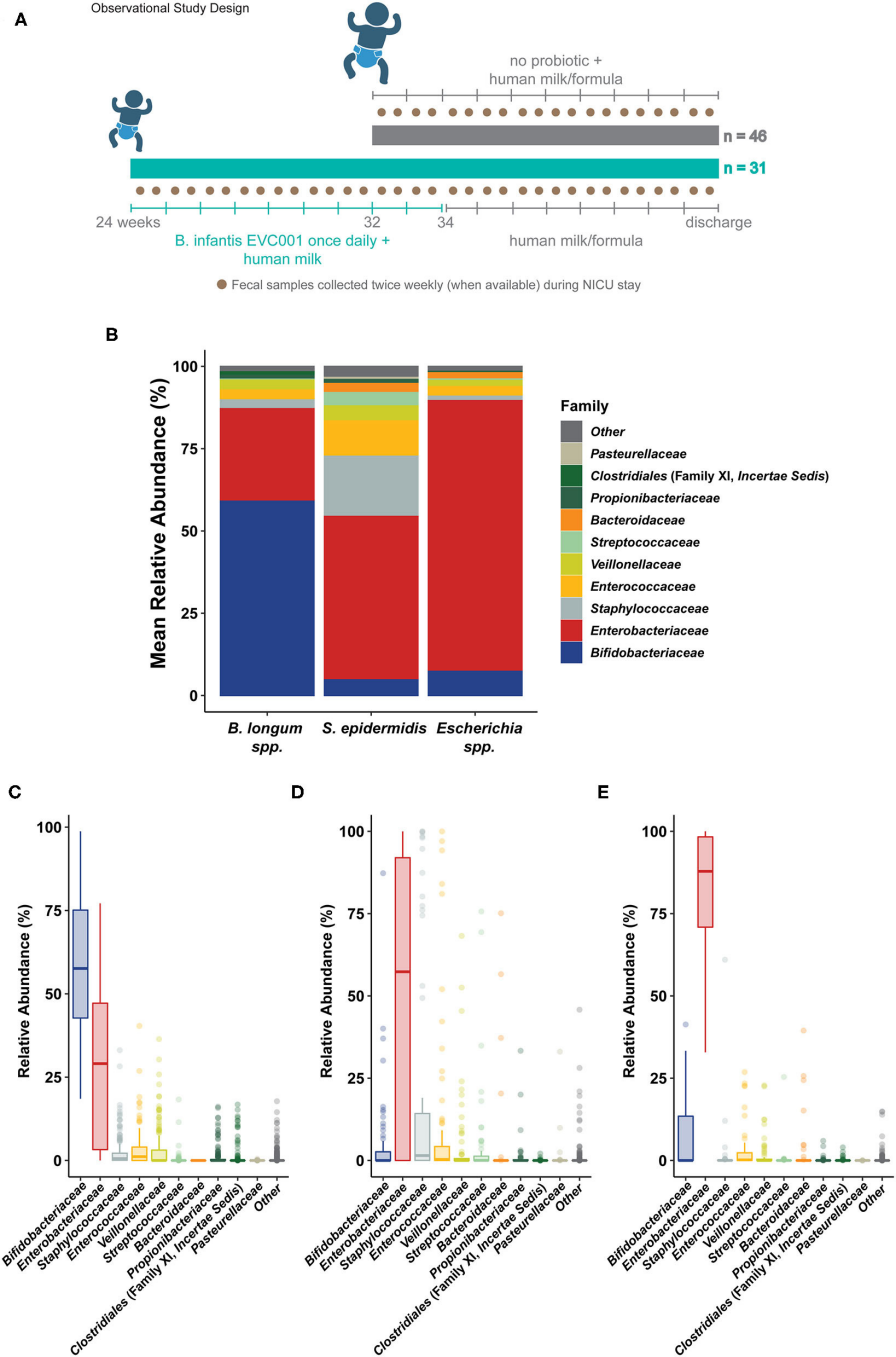
Sample collection and general sample microbiome composition. **(A)** The general study design is outlined describing the patient cohorts, per hospital feeding protocol, and prospective fecal sampling. Infants born in two hospitals were enrolled shortly after birth and, per hospital protocol, assigned to a feeding protocol based on gestational age at birth and weight at birth. Infants born at >32 weeks corrected gestational age (cGA) and 1500 g were assigned to a feeding protocol which did not include a probiotic (gray, top). Infants born at <32 weeks cGA and/or <1500 g were assigned to a feeding protocol which included daily feedings of *B. longum* subsp. *infantis* EVC001 (8 × 10^9^ CFU once per day) starting with the initiation of trophic feeding (teal, bottom) and delivered in a single serving of 0.5 mL medium chain triglyceride oil. Nurses collected fecal samples (brown circles) opportunistically throughout the patient's stay in the neonatal intensive care unit (NICU), aiming for two or more samples per week. Infants received human milk (maternal or donor) through 34 weeks corrected gestational age, as well as human milk-based human milk fortifier. After 34 weeks, infant formula was used in addition to human milk. **(B)** Shotgun metagenome sequencing performed on samples collected from all 77 infants was used to classify the average community structure of the infant microbiome in all samples. Individual sample compositions were generally similar and typified by either: **(C)** a high abundance of *Bifidobacterium longum* species (inclusive of *B. longum* subsp. *infantis*; *Bifidobacteriaceae*, blue), **(D)** a high abundance of *Staphylococcus epidermidis* (*Staphylococcaceae*, gray) and *Enterobacteriaceae* (red), or **(E)** an overwhelming abundance of *Escherichia coli* and unclassified *Escherichia* (*Enterobacteriaceae*, red).

To examine the effect of feeding *B. infantis* EVC001 to a preterm infant population, we prospectively and longitudinally collected fecal samples from 77 infants born before 37 weeks postmenstrual age and compared the gut microbial composition and development, as well as enteric inflammation profiles. These infants were assigned to one of two distinct, hospital-directed feeding protocols based on gestational age at birth and weight at birth. One feeding protocol dictating daily feedings of *B. infantis* EVC001 (EVC001-fed; < 32 weeks corrected gestational age and/or <1500 grams; *n* = 31) and a second feeding protocol were used for premature infants born after 32 weeks corrected gestational age and > 1500 g at birth (“No probiotic”; *n* = 46; Figure 1A). All infants were predominantly fed human milk but were discordant for probiotic use based on their corrected gestational age. Further, we collected deidentified patient metadata to compare the impact of other clinical interventions on the preterm infant gut microbiome and to account for known differences between the feeding cohorts. Here, we find that EVC001-fed premature infants had increased colonization of *Bifidobacterium* in their gut microbiome, an increase in genes conferring efficient utilization of HMOs, significantly decreased overall abundance of antibiotic-resistant (bacterial) genomes (ARGs), and importantly, a decreased enteric inflammatory profile compared to premature infants not fed a probiotic, after accounting for other confounding clinical variables.

## Results

### Demographics and Clinical Observations of the Preterm Infant Subjects

Seventy-seven [77] preterm infants were enrolled from May to October 2019 from Kaiser Permanente Orange County Anaheim Medical Center (Anaheim, CA USA; Level 3 NICU, 25 beds) and Kaiser Permanente Orange County Irvine Medical Center (Irvine, CA USA; Level 2 NICU, 10 beds). No changes to the hospital standard of care were introduced by participation in this prospective sampling study. Participant demographic and clinical data are provided in Table 1. Per hospital feeding protocols, infants born at <32 weeks gestational age (GA) or at <1500 grams birth weight received *B. infantis* EVC001 with MCT oil, via orogastric or nasogastric tube starting at the initiation of trophic feeds and continued daily through 34 weeks corrected gestational age (cGA, *n* = 31). Infants born after 32 weeks GA did not receive *B. infantis* EVC001 or any other probiotic (*n* = 46; Figure 1A). Thus, the two study groups differed in general clinical acuity, in both feeding protocols and the level of acuity between hospitals. Given the differences in gestational age (*P* < 0.001), there was also a corresponding difference in birth weight (*P* < 0.001), but both groups received a predominantly human milk diet via maternal, donor, and/or commercial human milk through 34 weeks cGA and the infants born at an older GA not fed probiotics received more infant formula as a result (*P* < 0.001). Also, significantly more fecal samples were collected from the infants fed *B. infantis* EVC001 compared to infants not fed probiotics [6.23 vs. 2.26 on average, *P* < 0.001] due to the generally longer duration of stay of the infants born more premature, and infants born at an earlier gestational age received more inhaled steroids, owing to their prematurity (e.g., albuterol, budesonide, levalbuterol, ipratropium; 22 vs. 0.9%, *P* < 0.001). Feeding *B. infantis* EVC001 was well tolerated, and no adverse events related to probiotic consumption were observed.

**Table 1:** Characteristics of preterm infant feeding cohort and sample collection.

**Sample and patient demographics**	**No probiotic**	***B. infantis* EVC001**	***P* value**	**Statistical comparison**
Total	46	31		
Female, *n* (%)	23 (50)	10 (32)	0.1651	Fisher's exact test
Gestational age, wk, mea *n* (SD)	34.9 (2.74)	28.3 (2.77)	< 0.001	Mann–Whitney *U*-test
Birth weight, g, mea *n* (SD)	2207 (738)	1112 (340)	< 0.001	Mann–Whitney *U*-test
Cesarean delivery, *n* (%)	31 (67)	20 (65)	1	Fisher's exact test
Fecal sample collected, *n*:	104	193		
Specimens collected per baby, mea *n* (SD)	2.26 (1.71)	6.23 (3.35)	< 0.001	Mann–Whitney *U*-test
Antibiotic exposure within 7 days of sampling, *n* (%)	14 (13.5)	10 (5.2)	0.024	Fisher's exact test
Infant formula use coinciding with sampling, *n* (%)	39 (37.5)	9 (4.66)	< 0.001	Fisher's exact test
Diaper rash reported coinciding with sampling, *n* (%)	14 (13.5)	10 (5.2)	0.024	Fisher's exact test
Steroid use coinciding with sampling, *n* (%)	1 (0.96)	43 (22.28)	< 0.001	Fisher's exact test
Fecal sample sequencing passing quality filter, *n* (%)	101 (97)	191 (99)		
Fecal sample inflammatory quantification, *n* (%)	83 (80)	171 (89)		

### Alteration of Microbiome Composition in Preterm Infants Fed *B. infantis* EVC001

**Figure 2:**
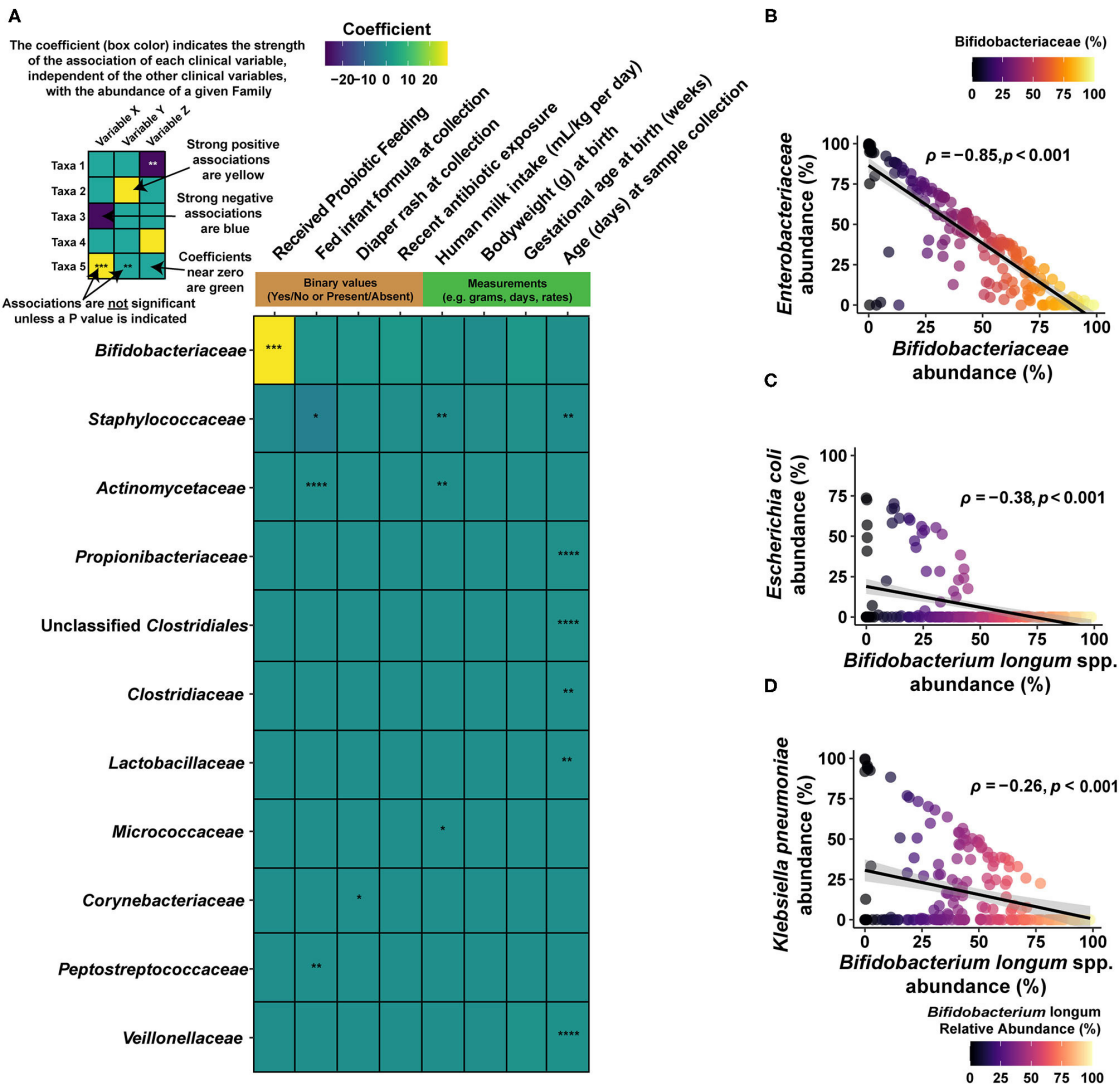
Associations between gut taxa and clinical interventions. **(A)** Heat map describing the output of multivariate analysis of individual clinical variables (columns) with family-level microbial groups. This approach separates the effects of individual clinical variables and reports the impact of each one, individually, on the abundance of the given bacterial family (row). Significant associations are indicated by the notation of the FDR-adjusted *P* value (asterisks), and the degree of the interaction (coefficient) is indicated by the color scale. Strong, positive associations between a given clinical variable (column) and the bacterial family (row) are indicated in yellow, while strong negative associations between a clinical variable and a bacterial family are indicated by dark blue. Weak associations (i.e., close to 0) are indicated in green. Only bacterial families that included a significant association prior to FDR correction are shown. As *Bifidobacteriaceae* are strongly linked to probiotic intervention (as indicated by the yellow tile), we examined interactions between this family and its constituents and other bacterial groups among infants fed *B. infantis* EVC001. **(B)** At the family level, *Bifidobacteriaceae* are strongly negatively correlated with *Enterobacteriaceae*, as assessed using relative abundance and Spearman's rho (ρ), among infants fed *B. infantis* EVC001. Points are shaded based on the abundance of *Bifidobacteriaceae*. This is also true for two of the primary constituents of the *Enterobacteriaceae* family. **(C)** *E. coli* and *B. longum* species are negatively correlated among infants fed *B. infantis* EVC001. **(D)** *B. longum* is negatively correlated with *K. pneumoniae* among infants fed *B. infantis* EVC001 (FDR-adjusted *P* values are indicated by asterisks, ****P < *0.0001*; ***P < *0.001*; **P < *0.01*; *P < *0.05*).

Shotgun metagenomic sequencing was performed on 292 samples, yielding an average cluster of 33,658,645 (± SD 7,269,047.16) and totaling 67,317,290 (± SD 14,538,094.3) reads per sample. After quality-filtering and removal of reads mapping to the human genome using GenCOF (31), 33.6 million sequences per sample (±7.8 million, SD) were processed for microbial taxonomy and functional classification. Using a cross-validated clustering approach, three broad compositional cluster types were identified (Figure 1B; see Methods). Typical communities in these cluster types were predominantly composed of either *Bifidobacterium longum* species (which includes *B. infantis*; 56.91%, +/− 20.97% SD), *Escherichia coli* (56.14%, +/− 16.00% SD), and unclassified *Escherichia* (19.11%, +/− 6.88%), or a more variable mix of taxa that included *Staphylococcus epidermidis* (14.22%, +/− 27.51% SD), *Klebsiella pneumoniae* (23.54% +/− 37.15% SD), and *Enterococcus faecalis* (10.73%, +/− 24.86% SD; Figure 1B). Congruent with the higher sample frequency from infants fed *B. infantis* EVC001, the majority of the samples fell into the high *B. longum* species cluster type (*n* = 160), with other samples split between the *Escherichia* cluster type (*n* = 63) and the more variable cluster type characterized by *S. epidermidis* (*n* = 69). Samples from infants fed *B. infantis* EVC001 were not evenly distributed among the cluster types, with 90% of samples from the *B. longum* species cluster having originated from infants fed *B. infantis* EVC001, and only 37 and 33% of samples from the *Escherichia* species cluster and mixed *S. epidermidis* cluster were from infants fed *B. infantis* EVC001, respectively. In order to test for the overall diversity between the three sample clusters and their effect size on the observed clusters, we ran a permutational multivariate analysis of variance using distance matrices (adonis) obtained from the sample clustering analysis. The adonis result by cluster assignment was significant (*P* = 0.001), suggesting that the three observed clusters were different and the effect size of the observed clustering is strong (R^2^ = 0.42). Alpha diversity was computed to estimate differences in microbial community composition in terms of observed bacterial species and community entropy, using the Shannon Diversity Index. A linear mixed model was used to account for clinical and demographic covariates as well as within-subject variations over time (see Methods). The probiotic-fed group reported a slightly lower alpha diversity compared to the controls (*P* = 0.009; FDR). The complete results of the FDR-adjusted linear mixed model are reported in Supplemental Table 1, while individual Shannon diversity indices are reported in Supplemental Table 2. In order to account for clinical variables associated with prematurity, and which differed between infants assigned to each feeding protocol, we used a boosted, additive linear mixed-effects model (MaAsLin2; https://huttenhower.sph.harvard.edu/maaslin/) to independently assess the effect that each of these clinical variables had on the gut microbiome. When accounting for the individual effect of clinical variables (i.e., probiotic feeding, inhaled steroid use, diaper rash, recent antibiotic exposure, diet, total parenteral nutrition (TPN) intake, age at sampling, weight at birth, gestational age at birth), we found that while several of them (diaper rash, infant formula in diet, human milk in diet, age at sampling) did have small, but significant associations with various bacterial families, the size of the effect of these clinical variables was quite low when compared to probiotic feeding (Figure 2A). Notably, the introduction of *B. infantis* EVC001, independent of other clinical variables, was associated with 27.6% more *Bifidobacteriaceae* abundance, when all other clinical variables were controlled (FDR-adjusted *P* value = 0.000183; Figure 2A). Second, while formula feeding (any amount) was associated with less *Staphylococcaceae* (−3.65%, FDR-adjusted *P* value = 0.0112), other associations included age at sampling (day of life), formula feeding, human milk feeding volume (mL/kg^*^day), and diaper rash, all of which were associated with significant differences in the gut microbiome (FDR-corrected *P* < 0.05), but these associations accounted for less than a 1% change per unit increase (e.g., mL/kg^*^day, week, or day, as indicated; Figure 2A). We also found that delivery mode had no significant associations with the microbiome, when the other clinical variables were considered.

As inclusion of feeding *B. infantis* EVC001 into the diet of infants had the largest individual impact on gut microbiome composition (Figure 2A), we also sought to understand how *Bifidobacteriaceae* abundance interacted with other taxonomic groups among infants fed EVC001. Broadly, increased *Bifidobacteriaceae* abundance was strongly associated with diminished *Enterobacteriaceae* abundance (which includes the *Klebsiella* and *Escherichia* genera; Spearman's ρ = −0.85, *P* < 0.001), and species-level discrimination confirmed this association between *B. longum* species (inclusive of *B. infantis*) and the most predominant *Enterobacteriaceae* species, *Escherichia coli*, and *Klebsiella pneumoniae*, with significant negative correlations between *B. longum* species and each of two *Enterobacteriaceae* species among infants fed *B. infantis* EVC001 (respectively, Spearman's ρ = −0.38, *P* < 0.001, Spearman's ρ = −0.26, *P* < 0.001; Figures 2C,D). This was further corroborated by comparing with species-specific qPCR for *B. infantis* with the abundance of these bacteria at different taxonomic levels (Supplementary Figure 1).

Given that this approach isolates the effect of each clinical variable on the infant gut microbiome from the others, we also explored how interactions between clinical variables and the gut microbiome were further interrelated. *B. infantis* is known to utilize human milk oligosaccharides as part of its colonization of the gut microbiome, so we examined whether a greater abundance *Bifidobacteriaceae* was associated with increased rates of human milk feeding. There was a significant correlation between the volume of human milk in an infant's diet and the abundance of *B. longum* species (Spearman's ρ = 0.26, *P* < 0.001), but only among infants who had been fed *B. infantis* EVC001 (Supplementary Figure 2A). Infants who were not assigned to the feeding protocol that included *B. infantis* EVC001 did not have a significant association between human milk feeding volume and the abundance of *Bifidobacteriaceae* (Supplementary Figure 2B; Spearman's ρ = 0.046, *P* = 0.649). The magnitude and relationship between *B. longum* species and human milk feeding volume only among infants fed EVC001 was confirmed by subspecies-specific qPCR for *B. infantis* (Spearman's ρ = 0.34, *P* < 0.001; Supplementary Figures 2C,D).

### Gut Microbiome Development Is Distinct in Infants Fed EVC001

As we identified limited impacts of differing clinical variables between the two feeding cohorts apart from probiotic use and the associated increased abundance of *Bifidobacteriaceae* (i.e., *B. infantis*; Figure 2A), which was associated with diminished abundance of other taxa typical for the preterm infant gut microbiome (Figures 2B–D), we examined the microbiome composition of infants in both feeding cohorts over time. Fecal samples from EVC001-fed infants developed a gut microbiome distinct from preterm infants not fed a probiotic. In addition to distinct functional differences, overall taxonomic composition differed, though a wider range of *B. infantis* relative abundances were observed when compared to previous studies examining the organism in healthy breastfed infants born at term (27). Taxonomic classification across all samples identified variable community compositions predominantly composed of *Escherichia* [32.59% (+/− 40.00% SD) vs. 9.95% (+/− 23.58% SD)], *Klebsiella* [12.09% (+/− 25.61% SD) vs. 22.52% (+/− 28.35 SD)], *Bifidobacterium* [11.50% (+/− 24.10% SD) vs. 47.50% (+/− 27.59% SD)], *Staphylococcus* [11.35% (+/− 27.07% SD) vs. 3.24% (+/− 9.53%)], *Enterobacter* [4.64% (+/− 16.28% SD) vs. 5.25% (+/− 15.72% SD)], and *Enterococcus* [7.73% (+/− 17.76% SD) vs. 3.29% (+/− 10.02% SD)] when comparing samples from infants not fed *B. infantis* EVC001 to infants fed the probiotic.

**Figure 3:**
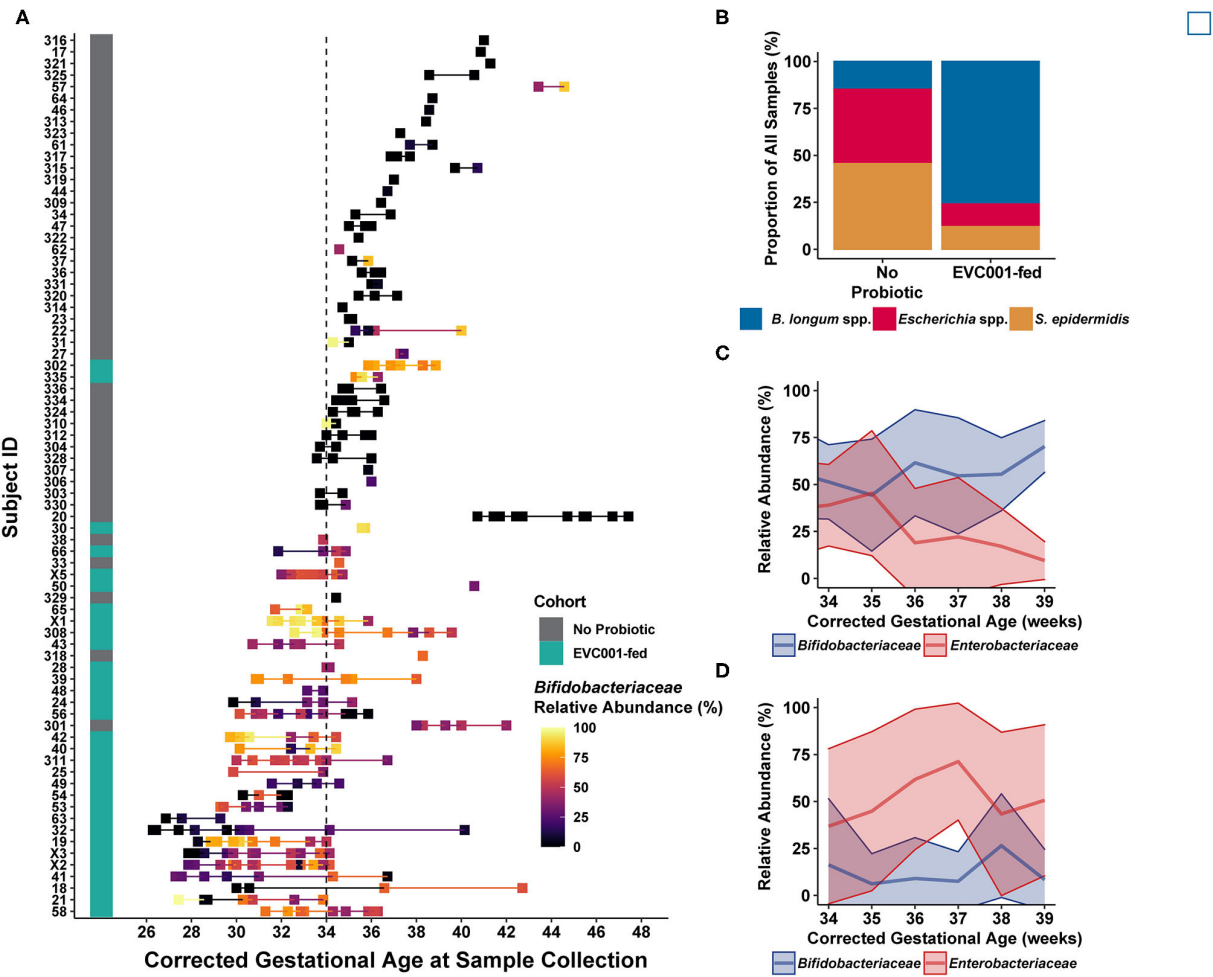
Infant fecal samples collected over time reflect feeding protocols and improved development trajectories with *B. infantis* EVC001. **(A)** Infant fecal samples (squares) collected over time, arranged by individual patient (y-axis) and corrected gestational age at sample collection, and ordered by gestational age of the infant at birth (from earliest gestational age at birth, bottom, to oldest gestational age at birth, top) and shaded by the abundance of *Bifidobacteriaceae* (see color scale). Infant feeding protocol is denoted by the teal (EVC001-fed) or gray box (No Probiotic). Note that the feeding protocol assignment is based both on gestational age at birth and birth weight, so some subjects (e.g., 302, 335) were assigned to the probiotic feeding protocol based on weight at birth (i.e., < 1500 g). **(B)** Fecal sample composition was clustered by species-level signature taxa (e.g., *B longum* species, *Escherichia coli* and unclassified *Escherichia*, and *S. epidermidis*), as indicated by color. Infants in the EVC001-fed feeding protocol more frequently developed a gut microbiome composed of primarily *B. longum* species (blue), while the infants not assigned to receive the probiotic frequently had compositions typified by high levels of *Enterobacteriaceae* (e.g., *Escherichia* species) and/or *Staphylococcus* (red and orange, respectively). **(C)** Over time, infants fed *B. infantis* EVC001 developed a high mean abundance of *Bifidobacteriaceae* (blue line) which increased as infants approached term gestational age, and this was negatively associated with the abundance of *Enterobacteriaceae* (red line). **(D)** In contrast, among infants not fed *B. infantis* EVC001, the abundance of *Enterobacteriaceae* did not change as infants approached term gestational age.

We found that infants fed *B. infantis* EVC001 typically developed a high relative abundance of *Bifidobacteriaceae*, while infants who were not fed *B. infantis* EVC001 rarely acquired high levels of *Bifidobacteriaceae*, despite human milk feeding (Figure 3A). Notably, infants who were <1500 g at birth were assigned to the feeding protocol which included *B. infantis* EVC001 and these infants also developed a high abundance of *B. infantis* (Subjects 302 and 335; Figure 3A). Accordingly, the species cluster identity of samples from infants fed *B. infantis* EVC001 were predominantly composed (76%) of the *B. longum* species cluster (Figure 3B) and this increased as infants reached maximum human milk feeding volumes (33.8 weeks median corrected gestational age) to 81%. In contrast, samples from infants not fed *B. infantis* EVC001 were primarily identified as belonging to the *Escherichia* species or *S. epidermidis* species clusters by the time they reached the maximum human milk feeding volumes (38 and 49%, respectively, at 36.1 weeks median corrected gestational age), and this was consistent with the sample identity overall from infants not fed *B. infantis* EVC001, representing 40 and 46% of samples collected from these infants, respectively (Figure 3B). We corroborated these findings by examining the absolute abundance of *B. infantis* via species-specific qPCR (see Methods). Similar to results obtained by metagenomic sequencing, we found that among infants fed *B. infantis* EVC001, *B. infantis* levels rapidly increased over time (Supplementary Figure 3) and reached maximum levels, in terms of both absolute and relative abundance, as infants approached discharge from the hospital (Supplementary Figure 3).

### *B. infantis* EVC001 Feeding Improves Functional Capacity of Preterm Infant Gut Microbiomes for HMO Utilization

**Figure 4:**
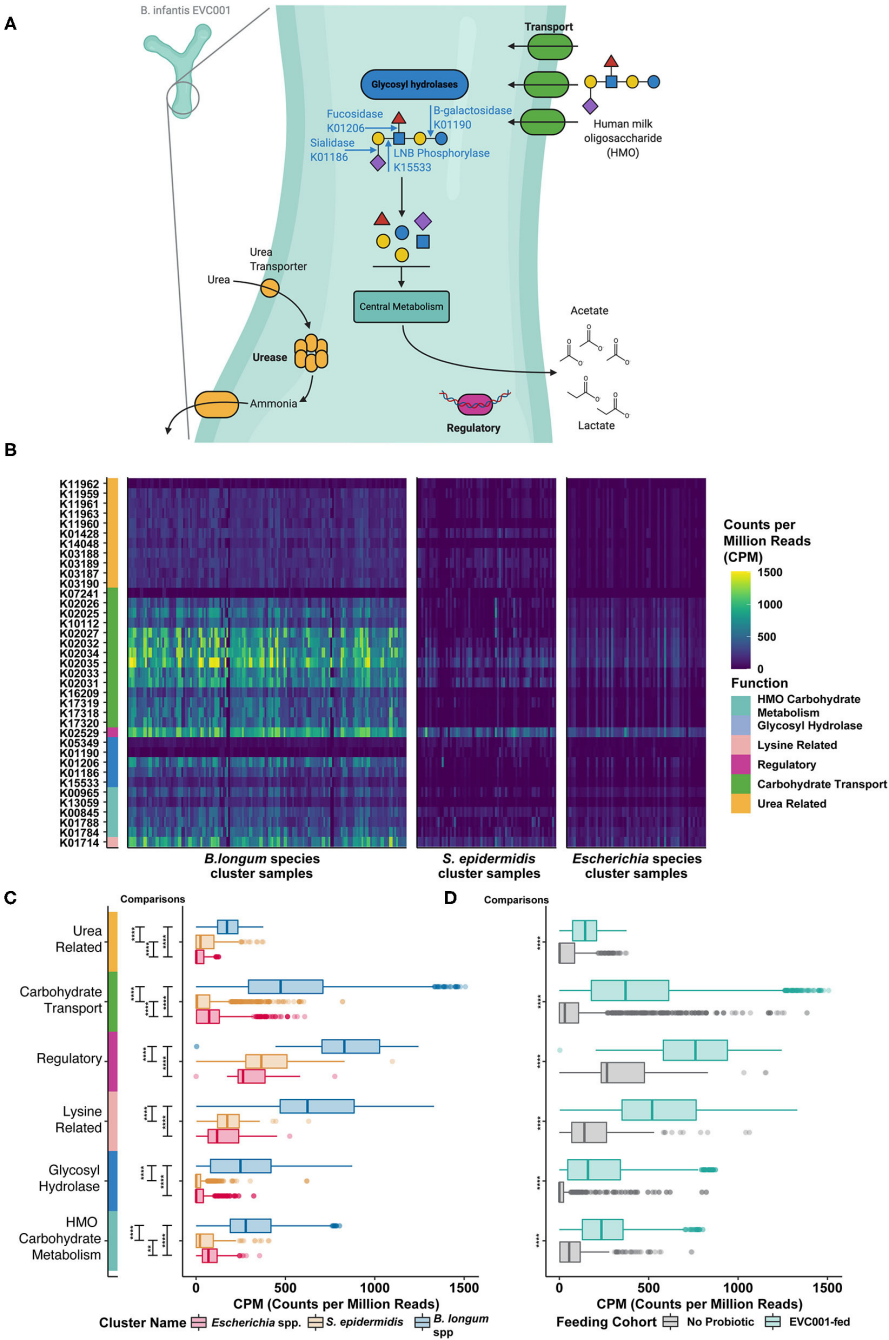
Human milk oligosaccharide (HMO) utilization in *B. infantis* and the enrichment of HMO-utilizing functions among infants fed *B. infantis* EVC001. **(A)** *Bifidobacterium longum* subsp. *infantis* (*B. infantis*) utilizes a suite of human milk oligosaccharide (HMO)-associated gene loci to transport intact HMO structures into the cell, where intracellularly located glycosyl hydrolases (blue) break down linkages found commonly among HMO structures. Other enzymes involved in central carbohydrate metabolism (teal) are used to convert sugars to the primary metabolic outputs, acetate, and lactate. Other features associated with HMO utilization include regulatory elements (purple) and urea metabolism (orange). **(B)** These functions, which are represented as distinct KEGG orthologs, are differentially abundant among samples, depending on the predominant bacterial species. Samples (columns) with greater abundance (i.e., higher CPM, counts per million) of HMO-associated KEGG orthology (rows) were found among the *B. longum* species cluster. **(C)** All six functional categories were significantly enriched (i.e., greater CPM) in samples from the *B. longum* species group (blue box plot), relative to samples composed primarily of *S. epidermidis* (orange box plot) or *Escherichia* spp. (red box plot). This difference is associated with probiotic *B. infantis* EVC001 feeding **(D)** as these functional categories were enriched among samples from infants fed EVC001, relative to samples from infants who were not (*P* values, adjusted for multiple comparisons, are indicated by asterisks, ****P < *0.0001*; ***P < *0.001*; **P < *0.01*; *P < 0.05). Image created with BioRender.com.

Given that infants in the study population were fed human milk through 34 weeks cGA or longer, with or without human milk-based fortification and/or formula, we conducted an exploratory analysis using the KEGG Orthology (KO) database of functional orthologs to identify 37 representative key functions necessary to metabolize human milk oligosaccharides (e.g., oligosaccharide binding proteins, sialidases, fucosidases, lacto-N biosidase, etc.) identified by Sela et al. (32) (Figure 4A). We then compared the distribution of these functions across each of the sample clusters and examined the impact of *B. infantis* EVC001-feeding on the abundance of these functions. A comparative analysis indicated that the relative amount (counts per million, CPM) of KO functions related to HMO metabolism was globally enriched among samples from infants that belonged to the *B. longum* species cluster (Figure 4B).

In *B. infantis*, glycolytic enzymes are internally localized and require complex HMO-transport systems (e.g., ABC transporters; green, Figure 4A) to facilitate efficient uptake of HMO structures into the bacterial cell (33). Functions in this category were significantly more abundant among samples from EVC001-fed and from samples belonging to the *B. longum*-species cluster (Figures 4B,C). While some *Bifidobacterium* species are able to externally degrade carbohydrates and transport the constituent monomers and dimers [e.g., *B. bifidum* (34)] and others are only able to access HMOs by the glycolytic activity of other bacteria [e.g., *B. breve* (35)], the mean abundance of these *Bifidobacterium* among these samples was very low (1.7% *B. breve*, 0.142% *B. dentium*, and others below 0.01%) and rare (only three subjects had > 30% *B. breve* at any time, only one had > 30% *B. dentium*). These data emphasized both the rarity of *Bifidobacterium* among NICU patients absent probiotic intervention and the relative importance of the carbohydrate transport systems to enable HMO utilization by *Bifidobacterium* present in these samples.

Among the functions necessary for HMO utilization by the gut microbiome, glycolytic enzymes including fucosidases and sialidases are indispensable for the primary removal of terminal fucose and sialic acid moieties from complex HMO molecules (blue, Figure 4A). These functions enable degradation of intact HMO structures into their constituent fragments and the transfer of these fragments to fermentation pathways (Figure 4A). Glycolytic functions were significantly enriched among infants in the *B. longum* species cluster and among EVC001-fed infants in other cluster types (teal, Figure 4A) as were key biosynthetic steps in the cleavage of core I and core II HMO glycans, lacto-*N*-biose, and *N*-acetyllactosamine (e.g., K013509) via sugar kinases, as well as utilization of sialic acid (e.g., K01788) and *N*-acetyl-hexoses (e.g., K15533) (Figure 4C). Together, these results demonstrate greater primary HMO degradation potential among infants colonized by *B. longum* species (Figure 4C), and particularly, among EVC001-fed infants as all general functions were significantly enriched (*P* < 0.0001) among these samples (Figure 4D).

*B. infantis* also has genes enabling the transport of urea and the hydrolysis of urea to ammonia co-localized in the genome with HMO metabolism (32). As human milk naturally contains urea (45 μmol/L) (36), the liberation of ammonia from urea as a nitrogen source has been proposed for *B. infantis* (32). Genes involved in urea metabolism (urea transport and hydrolysis; orange, Figure 4A) were significantly enriched among samples from infants belonging to the *B. longum* species cluster type (Figure 4B), and among EVC001-fed infants (Figure 4C). Samples from infants that were classified in the mixed *S. epidermidis* cluster type were also enriched for urease transport and activity, relative to the *Escherichia* species cluster type. Among the Gram-positive organisms, which were comparatively enriched among these samples, urease activity is chiefly thought to be a pH-buffering mechanism (37).

Other functions identified in *B. infantis* HMO-related gene clusters which included genes related to regulatory functions [e.g., a lacI homolog, purple, K02529; and a protein involved in lysine metabolism (4-hydroxy-tetrahydrodipicolinate synthase), pink, K01714] were also significantly more abundant (*P* < 0.0001) among samples from infants in the *B. longum* species group and among samples from infants fed *B. infantis* EVC001 (Figures 4C,D).

### Antibiotic Resistance Genes Are Acquired During NICU Stays and Differ Between Hospital NICUs

**Figure 5:**
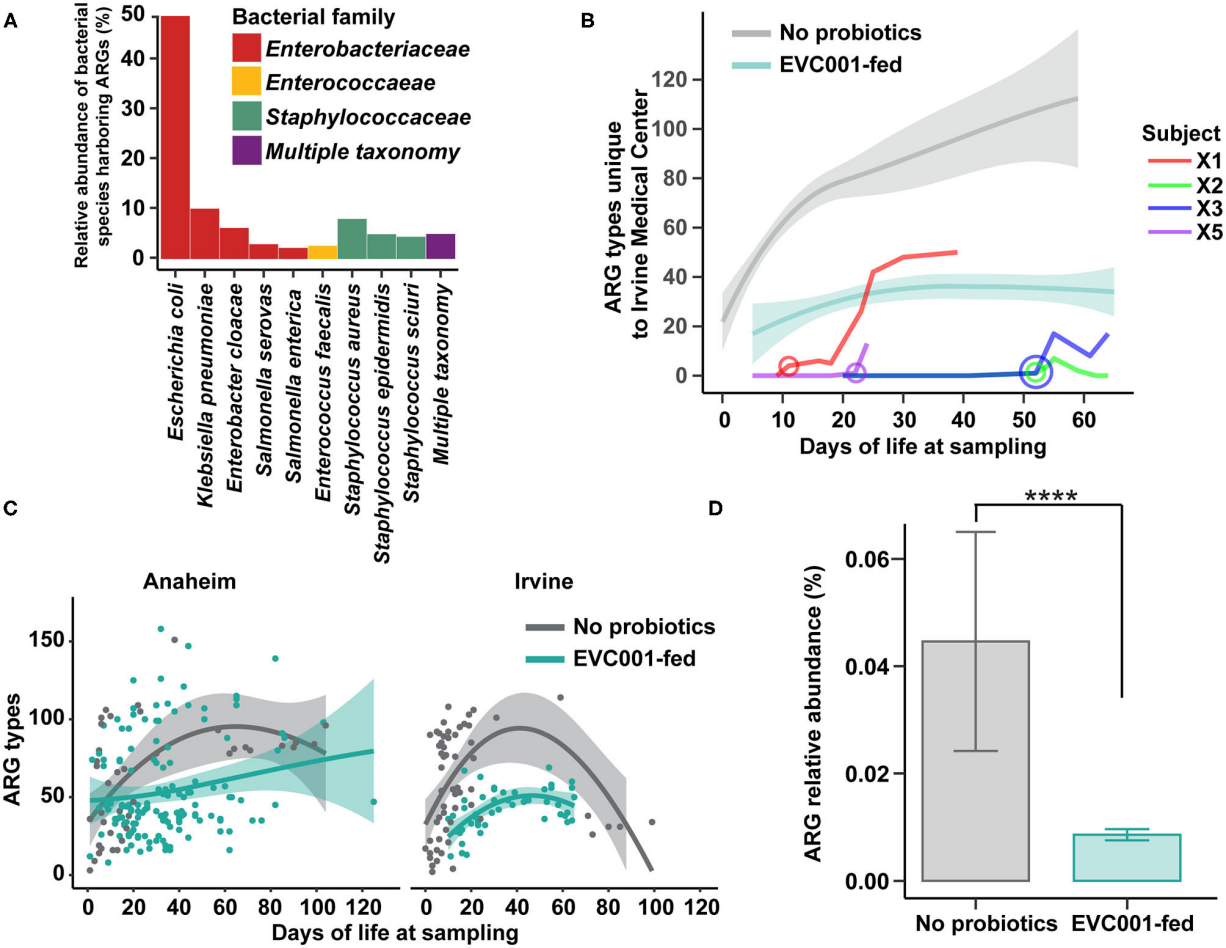
Antibiotic resistant bacteria and antibiotic resistance genes are reduced among infants fed *B. infantis* EVC001, irrespective of hospital location. **(A)** The relative proportion of antibiotic resistance genes identified among taxa in fecal metagenomes. Nearly all ARGs could be assigned to *Enterobacteriaceae* (red), *Enterococcaceae* (yellow), or *Staphylococcaceae* (green), though some could not be confidently assigned to one organism (purple). **(B)** Infants transferred between hospitals participating in this study demonstrated rapid colonization by bacteria harboring hospital-specific ARGs after their transfer date (circle, age in days at transfer). All four transferred infants were assigned to the feeding protocol including *B. infantis* EVC001, and the amount of hospital-specific ARGs trended lower and similar to other infants in the unit that received *B. infantis* EVC001 (teal line, confidence intervals), relative to infants who did not receive the probiotic (gray line, confidence intervals). **(C)** Infants at the two hospitals developed similar patterns of ARG colonization, with more than 100 different unique ARGs detected. Infants acquired ARGs throughout their stay, though infants fed *B. infantis* EVC001 tended to acquire fewer unique ARGs (teal), relative to infants who did not receive the probiotic (gray). **(D)** Samples from infants fed *B. infantis* EVC001 also exhibited a lower overall relative abundance of ARGs (teal), as a proportion of their microbiome, compared to samples from infants who did not receive the probiotic (gray) (*P* values indicated by asterisks, ****P < *0.0001*).

As the spread of antibiotic-resistant bacteria is of particular concern in clinical practice, we examined the abundance of antibiotic resistance genes (ARGs) in samples collected from infants in this study. We first profiled the taxonomic composition of ARGs in all stool samples (i.e., the resistome). Nine species were responsible for carrying 86.2% of the total ARGs identified in the study, while 4.5% of ARGs could not be confidently assigned to a single species (Figure 5A). The nine species belonged to the families of *Enterobacteriaceae, Enterococcaceae*, and *Staphylococcaceae. Escherichia coli* harbored 48.5% of the total resistome, followed by *Klebsiella pneumonia* (9.6%), *Staphylococcus aureus* (7.5%), and *Enterobacter cloacae* (5.7%). No significant differences in the relative abundance of bacterial species harboring ARGs were found between the hospitals studied here (Figure 5A).

Next, we investigated unique ARGs in each NICU and found that of the 315 genes identified, 204 were common in both units, while 19 ARGs were specific to Irvine Medical Center and 92 were specific to the Anaheim Medical Center (Supplementary Table ARG 3). However, only the abundance of four ARGs was significantly different when accounting for sample distribution between hospitals. Specifically, the abundance of *aadA5* (ARO:3002605), an aminoglycoside nucleotidyltransferase known to confer resistance to streptomycin/spectinomycin and encoded by plasmids, transposons, and integrons in *E. coli, K. pneumoniae, P. aeruginosa*, and *E. cloacae* (38), was significantly more abundant in the Irvine compared to the Anaheim NICU (*P* = 0.01, Bonferroni). The aminoglycoside resistance gene, *aph(2”)-IIIa* (ARO:3002636) (39), was also unique to the Irvine Medical Center. Conversely, the level of gene *AAC(6)-Ic* (ARO:3002549), known to confer aminoglycoside resistance in *Klebsiella ssp* (39), was significantly more abundant in the Anaheim compared to Irvine NICU (*P* = 0.03; Bonferroni). Finally, ARO: 3003291 was the only significantly different ARG found within both hospitals and was 1.2-fold higher in the Irvine NICU (*P* = 0.02; Bonferroni). This gene is known as *Staphylococcus aureus rpoC* conferring resistance to daptomycin, a lipopeptide antibiotic with potent activity against Gram-positive bacteria (40) (Supplementary Table ARG 3).

While there was not a difference in the taxonomic identity of ARGs between hospitals, we did identify ARGs specific to each hospital site (Supplementary Table ARG 3). Four *B. infantis* EVC001-fed infants were transferred from the Anaheim Medical Center to the Irvine Medical Center at different times throughout the duration of the sample collection period, and we found all four infants acquired unique, hospital-specific ARGs after transferring to the new hospital (Figure 5B; Supplementary Table ARG 4). Importantly, these four infants were colonized with novel hospital-specific ARGs acquiring 66 new ARGs, on average, in 48 days dependent on NICU length of stay. Importantly, these four transferred infants reached ARG levels comparable to infants under the same feeding protocol including *B. infantis* EVC001 and maintained fewer ARGs than infants on the feeding protocol that did not include *B. infantis* EVC001 (Figure 5B).

### Colonization by *B. infantis* EVC001 Is Associated With a Reduced Antibiotic-Resistant Gene Burden in Preterm Infants

The mean ARG load (i.e., the resistome) expressed in total percent composition of the metagenome identified against the CARD database was 0.04% in the group not fed the probiotic and 0.008% in the EVC001-fed infants, a difference in mean ARG abundance of 80.6% (*P* < 0.0001; Figure 5D). A total of 315 unique ARGs were identified among all samples from the CARD database (Supplementary Table ARG 5). Of those, 85 ARGs were differentially abundant between EVC001-fed infants and those who were not fed probiotics (*P* < 0.05; Bonferroni). Eighteen unique ARGs were significantly more abundant in the EVC001-fed (mean 1.21-fold higher) compared to no probiotic preterm infants (Supplementary Table ARG 6). Conversely, 67 unique ARGs were significantly more abundant (270-fold higher, on average) in the no probiotic compared to EVC001-fed preterm infants. In particular, three ARGs were several orders of magnitude lower in abundance among the samples from EVC001-fed infants compared to preterm infants not fed the probiotic. The gene *cat* (ARO:3002670) was 10^4^-fold less abundant (*P* = 0.005; Bonferroni) in EVC001-fed compared to infants not fed a probiotic. *Cat* is a well-known chloramphenicol acetyltransferase gene described in a range of bacteria including *Enterococcus* and *Staphylococcus* spp. conferring resistance to phenicol antibiotics (41, 42). *Mpha* (ARO:3000316) is a gene that encodes for the resistance enzyme MPH (2')-I, known to inactivate multiple macrolides (e.g., azithromycin, erythromycin) in species including *Enterobacter cloacae, Escherichia coli, Klebsiella oxytoca*, and *Klebsiella pneumoniae. Mpha* was found to be 4.6 × 10^3^ fold lower among EVC001-fed compared to preterm infants not fed the probiotic (*P* = 0.01; Bonferroni). Of interest, only 10 preterm infants in the no probiotic group were known to have received erythromycin during their clinical stay. *Mrx* (ARO:3003839, recently updated as ARO:3000333) was 3 × 10^3^ fold lower in EVC001-fed compared to no probiotic preterm infants (*P* = 0.007; Bonferroni). *Mrx* is also associated with macrolide resistance and is part of the gene cluster *mphA*-*mrx*-*mphR*. The majority (70%) of the significant ARGs were classified as multidrug resistant. Forty-eight multidrug-ARGs were found to be on average 277-fold lower in EVC001-fed infants when compared to infants who were not fed the probiotic, which are known to confer resistance to several drug classes (14 different drug classes and up to 29 on average).

Finally, given infants in early life acquire microbes from the environment over time (Figure 5C), we built a mixed-effect linear model considering time as a function equal to the preterm infant's day of life when a sample was collected, respectively, to assess the rate of nosocomial ARG acquisition. We also considered whether feeding *B. infantis* EVC001 or the NICU location at the time of collection would have an impact on the model while controlling for individual subject variation. Our model predicted that with all other variables held constant in an approximate linear model, infants acquired on average a new ARG-type for every 2 days spent in the NICU (*P* < 0.0001). This finding was consistent between hospitals (*P* > 0.05). Among preterm infants fed *B. infantis* EVC001, there were 13.6 fewer ARG types, on average, compared to preterm infants who were not fed the probiotic throughout their entire NICU stay (*P* < 0.05).

### Significantly Decreased Enteric Inflammation in Low Birth Weight Premature Infants Fed *B. infantis* EVC001 Compared to Infants Born Closer to Term at the Same Gestational Age

**Figure 6:**
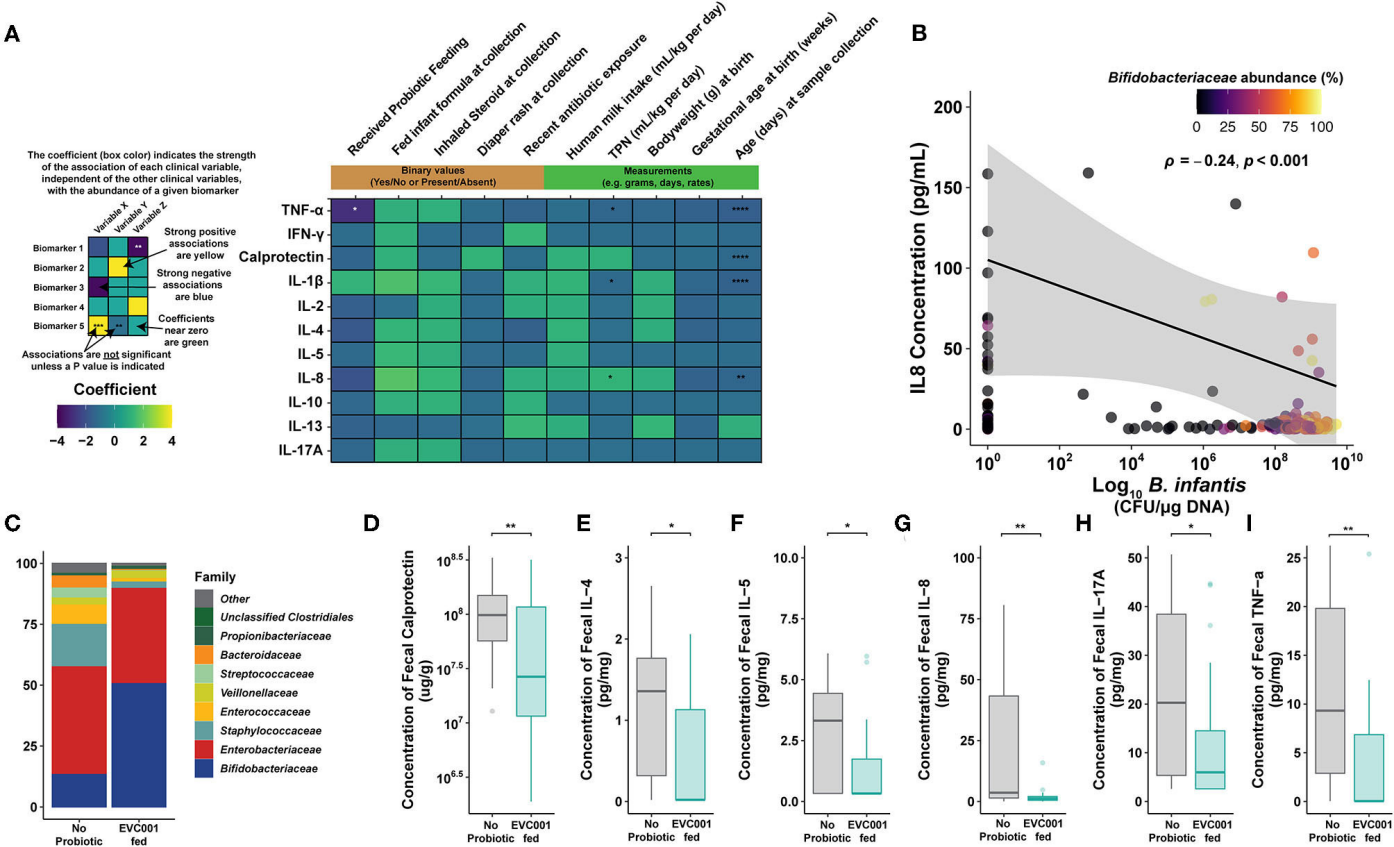
Proinflammatory biomarkers are significantly lower in preterm infants fed *B. infantis* EVC001. **(A)** Multivariate analysis by linear models (MaAsLin2) was used to assess the effect that each clinical variable had, independently, on proinflammatory biomarker concentrations. Probiotic feeding (*B. infantis* EVC001) was significantly associated with lower TNFα concentrations (-1.06 pg/mL per kg per day; FDR-corrected *P* value = 0.0011). TPN (mL/kg * day) correlated with increased IL-8 production (1.25 pg/mL per unit increase; FDR-corrected *P* value = 0.02) and a decrease of IL-1β and TNFα (0.064 and 0.1 pg/mL * day; FDR-corrected *P* value = 0.02, 0.02, respectively). Day of life at sampling associated with decreased calprotectin, IL-1β, IL-8, and TNFα production at a rate of 0.04, 0.07, 0.1, and 0.08 pg/mL * day, respectively (FDR-corrected *P* value = 0.0001, 0.0001, 0.002, and 0.0001, respectively). No significant associations between inhaled steroid exposure, diaper rash and treatment, antibiotic exposure, formula feeding, human milk consumption, birth weight, or gestational age at birth were identified. **(B)** Significantly decreased proinflammatory cytokine, IL-8, correlated with *B. infantis* EVC001 absolute abundance (*P* = *0.001*, ρ = −0.24). **(C)** The mean relative abundance of bacterial families in samples analyzed at the ~34-week time point, colored by family, is shown. Mean and standard deviations are available in Supplementary Table 7. **(D–I)** Box plots represent fecal calprotectin [ug/g] and proinflammatory cytokine concentrations [pg/mg] from infants not fed *B. infantis* EVC001 (*n* = 28) and EVC001-fed infants (*n* = 29) at 34 weeks corrected gestational age. Calprotectin and cytokine concentrations were measured in duplicate using MesoScale Discoveries R and U-plex, respectively. Statistical analysis was completed using Wilcoxon rank-sum test. *P-*values were adjusted using the FDR method and considered to be statistically significantly decreased if ****P < *0.0001;* ***P < *0.001;* **P < *0.01;* *P < *0.05*.

Inflammatory biomarker production, including cytokines and calprotectin, drives immunopathogenesis underlying the increased risk of morbidity and mortality in preterm infants (43, 44). Given our observational study design comparing EVC001-fed to no probiotic preterm infants, we first tested whether longitudinal cytokine and calprotectin concentrations were associated with clinical metadata or *B. infantis* EVC001 feeding using a multivariate analysis (Multivariate Analysis by Linear Models; MaAsLin, *n* = 245). Here, we showed that total parenteral nutrition (TPN) usage (mL/kg ^*^ day) significantly associated with increased IL-8 (Figure 6A; 0.07 pg/mL, FDR-adjusted *p* value, *q* = 0.018). Conversely, IL-1β and TNFα production decreased 0.064 and 0.1 pg/mL with increased TPN usage (mL/kg ^*^ day) (Figure 6A; FDR-adjusted *p* value, *q* = 0.018 and 0.018, respectively). Similarly, there was a weak negative relationship between calprotectin, IL-1β, IL-8, and TNFα concentration and age (in days) at a rate of 0.04, 0.07, 0.1, and 0.08 pg/mL per day, respectively (Figure 6A; FDR-adjusted *p* value, *q* = 0.0001, 0.0001, 0.002, and 0.0001, respectively). Notably, the strongest relationship was observed between decreased TNFα production with *B. infantis* EVC001 inclusion in the feeding protocol (Figure 6A; −1.06 pg/mL, FDR-adjusted *p* value, *q* = 0.01). Inhaled steroid usage, diaper rash, infant formula exposure, daily probiotic, birth weight (g), breast milk consumption (mL/kg per day), antibiotic exposure at sampling, and gestational age at birth (weeks) were not significantly associated with differences in cytokine and calprotectin concentrations when all other clinical variables were considered (Figure 6A). It is important to note the relationship between TPN and DOL with cytokine and calprotectin concentrations were weak and the coefficient of the relationship was below the level of detection for the assay. Further, we did not find a significant association between delivery mode and enteric inflammatory biomarkers when other clinical and demographic variables were considered.

Given the multivariate analysis identified *B. infantis* EVC001 feeding as the strongest association with decreased enteric inflammation, we sought to corroborate whether there was an association of *B. infantis* EVC001 with cytokine concentrations (IFNγ, IL-10, IL-13, IL-17A, IL-1β, IL-2, IL-4 IL-5, IL-8, and TNFα). *B. infantis* absolute abundance was negatively correlated with the production of IL-8 (Figure 6B; *P* = 0.001, ρ = −0.24) independent of microbiome cluster characterization and marginally negatively correlated with TNFα, though the latter was not significant (*P* = 0.055; ρ = −0.14).

Gut microbiome composition has previously been shown to modulate the enteric inflammatory markers in term and preterm infants (13, 19). Moreover, the hospital feeding protocol continued feeding of *B. infantis* EVC001 up to 34 weeks cGA (see Methods). Given that other clinical differences between the infants had small or nonsignificant associations with cytokine production, we next investigated whether EVC001-fed preterm infants had an altered enteric inflammation profile compared to no probiotic group at 34 weeks cGA. We selected samples collected from individual patients in each feeding group as close as possible to 34 weeks cGA and compared the compositional changes in the microbiome at this time point between the EVC001-fed (*n* = 29; mean cGA 34.1 weeks) and no probiotic preterm infants (*n* = 28; mean cGA 35.4 weeks). Among these samples, the only significantly different bacterial family was *Bifidobacteriaceae*, which was more abundant among samples from *B. infantis* EVC001-fed infants in this sampling window (Figure 6C; FDR-corrected *P* < 0.0001), though the microbiome composition of the control group was substantially more variable between subjects (Supplementary Table 7). The composition of cluster identity of the samples identified in the 34-week cGA sampling window was significantly different between the two treatment groups (χ^2^ *P* < 0.0001), with far more *S. epidermidis* cluster-associated samples among infants who did not receive the probiotic and more *B. longum* cluster-associated samples among infants fed *B. infantis* EVC001 (Supplementary Table 7). When considering samples from infants nearest 34 weeks cGA, significantly lower concentrations of calprotectin, IL-4, IL-5, IL-8, IL-17A, and TNFα (Figures 6D–I; FDR-corrected *P* = 0.009, 0.039, 0.026 0.009, 0.032, and 0.009, respectively) were observed among infants fed *B. infantis* EVC001 compared to infants not fed probiotics.

Taken together, these data showed that clinical metadata, including inhaled steroid use, diaper rash incidence, infant formula, daily probiotic, birth weight, breast milk consumption, antibiotic usage, and most importantly gestational age, were not associated with fecal cytokine or calprotectin concentration. However, the abundance of *B. infantis* EVC001, assessed by subspecies-specific qPCR, correlated with decreased enteric inflammation. Further, EVC001-fed preterm infants had significantly lower levels of key proinflammatory biomarkers compared to samples from infants not fed *B. infantis* EVC001, which suggests that colonization of the preterm infant gut microbiome by *B. infantis* EVC001 may help modulate enteric inflammation in premature infants.

## Discussion

The gut microbiome plays a major role in the development of the intestinal mucosa, including maturation of physiological, anatomical, and biochemical functions in infants (15). The intestinal microbiome composition and the infant's response to these colonizing bacteria are important risk factors for increased morbidity and mortality, including necrotizing enterocolitis (NEC) and sepsis in premature infants (24, 30, 45–47). Moreover, it is evident that premature infants born in hospitalized environments are colonized by nosocomially acquired bacteria, including antibiotic-resistant bacteria (6). This increases the risk of serious infection and death (5) and presents deleterious implications for preterm infant growth and development (48). While human milk feeding is an important component of the current strategy to mitigate these effects, our findings show that it is insufficient, alone, to prevent colonization by nosocomial pathogens or diminish the impact of these pathogens on the enteric immune system (19). Therefore, the importance of early microbiome development on host health and morbidity and the relative plasticity of this community presents a need, and an opportunity, to colonize the preterm gut microbiome with beneficial organisms that maximize ecosystem services to the host (26). These services include increased availability of nutrients from human milk, improved maturation of the intestinal epithelium, reduced enteric inflammation, and mitigated risk of infection in hospitalized infants (6, 24, 46, 47, 49). In this study, we found that preterm infants in two hospitals were colonized by nosocomially acquired bacteria despite a human milk diet, and the resulting gut microbiomes, absent intervention with *B. infantis* EVC001, developed comparably to reports in the literature on the preterm infant gut microbiome (4, 5, 13). Early acquisition of skin-derived bacteria (e.g., *Staphylococcus epidermidis*) and rapid colonization of the microbiome by *Enterobacteriaceae* are consistent with examples of preterm infant gut microbiome development in the literature (50, 51), with taxa-dependent implications for preterm infant morbidity and development (7, 13, 48). Among preterm infants fed EVC001, we observed that novel microbiome compositions developed, where colonization by *B. infantis* resulted in the displacement of bacteria associated with increased risk of preterm morbidities (8) (e.g., Figures 2B–D, 3). Moreover, when preterm infants were fed *B. infantis* EVC001, their overall microbiome was significantly enriched in functions required to access HMOs (32) (Figure 4). This is particularly relevant because the ability to utilize HMOs as prebiotic substrates enables *B. infantis* EVC001 to thrive and modify the biochemical environment by the production of lactate and acetate (27). Furthermore, the consumption of HMOs by *B. infantis*, an infant-adapted *Bifidobacterium*, produces critical immune modulatory metabolites, including indole-3-lactic acid (52). This functional adaptation to human milk also defines the symbiotic interaction between *B. infantis* and the human infant and is key to the delivery of critical microbiome ecosystem services important to health and nutrition (26). Epidemiological data have undeniably demonstrated that breast milk confers benefits for early and lifelong health to infants (53). However, the infant also relies on the gut microbiome to fully realize the benefits from breast milk, especially HMOs. We found that human milk feeding alone was not able to reduce the abundance of dysbiotic taxa (e.g., *E. coli*; Figure 2A). In contrast, our results showed that feeding *B. infantis* EVC001 was required to enrich the functions necessary to harvest additional energy from breast milk and limit populations of bacteria associated with dysbiosis and poor growth (e.g., *Klebsiella, Enterobacteriaceae*) (48) (Figures 2, 3).

Interestingly, we observed that EVC001-fed preterm infants had significantly less antibiotic exposure (Table 1) and significantly fewer incidences of diaper rash despite their relative prematurity at birth, which warrants further investigation to understand whether this was directly or indirectly related to colonization with *B. infantis* EVC001. Additionally, these infants had significantly fewer ARGs as a fraction of their microbiome, with a total reduction in the resistome of 80.6% compared to the no probiotic group. Specifically, multidrug resistance genes were, on average, 227-fold higher in infants not fed a probiotic and some samples had several thousand-fold higher abundance for specific ARGs compared to EVC001-fed infants, including *Mpha*, which confers resistance to macrolide antibiotics, including erythromycin and azithromycin (54). The majority of the ARGs identified in this study are known to be present in potentially pathogenic bacterial species, including *Klebsiella* and *Enterobacter* spp., which are responsible for nosocomial infections as well as more severe morbidities including NEC (55, 56) and late-onset sepsis (55, 56). In addition, we detected unique ARG signatures transferred to infants and were able to observe the rapid acquisition of site-specific ARGs, which highlights the NICU environment as a source of antibiotic resistance microbes that can be transferred between NICUs (57). We also found that infants acquire these ARGs throughout their NICU stay, suggesting that reducing hospitalization times may limit the acquisition of nosocomially acquired antibiotic-resistant bacteria. Importantly, targeted gut microbiome modulation using *B. infantis* EVC001 has now been shown to be an effective strategy to combat the spread of ARGs in both term (58) and preterm infants and is unlikely to lead to the development of novel resistance mechanisms.

Key *Bifidobacterium* species have been associated with normal development of immune tolerance, and *B. infantis*, in particular, has been shown to normalize the permeability of the intestinal mucosa (59, 60), which may partially explain why we saw a significant reduction in enteric cytokine production similar to previously reported findings in term infants (19). Indeed, we observed that EVC001-fed preterm infants had significantly lower enteric inflammation compared to preterm infants not fed a probiotic, which may be due in part to the reduction in the abundance of potentially pathogenic bacteria associated with higher levels of endotoxin (27). Alternatively, the absolute abundance of *B. infantis* EVC001, independent of microbiome composition, correlated with decreased proinflammatory cytokine profiles, which indicates a benefit in feeding *B. infantis* EVC001 and may support the hypothesis that *B. infantis*-derived bacterial metabolites, produced through the utilization of HMOs, induce mucosal immune tolerance in the gut (52, 61). Notably, *Bifidobacterium-*derived acetate has previously been shown to mitigate the pathogenesis of *Enterobacteriaceae* infection in an animal model (60), and *B. infantis-*derived indole-3-lactic acid production reduced pathogen-induced inflammation in enterocytes *in vitro* (52, 61) through activation of the aryl hydrocarbon receptor (52, 62). Recently, differences in *B. infantis* HMO utilization loci have been identified (63), and strains missing key genes involved in HMO utilization are unlikely to confer the same benefits to infants as we observed because they lack the ability to effeciently convert HMOs to infant accessible short-chain fatty acids and indole-3-lactic acid, which are key functions of a healthy gut microbiome, whether in term or preterm infants (26).

There are some limitations to the results presented here, chiefly by the observational design of the study. Equivalent longitudinal data sets from both cohorts, those not receiving probiotics, and those fed *B. infantis* EVC001 at similar cGAs were not available with which to compare our findings throughout the duration of their hospital stays. Given these potential confounding variables in our data set, we compared the effect of those differences in clinical variables on the gut microbiome and cytokine profile using MaAsLin, between EVC001-fed infants and infants who were not fed a probiotic (Figures 2A, 6A). We found that these differences in gestational age or weight at birth are unlikely to be responsible for the significant changes we observed between the feeding cohorts, given that the strongest effect was related to probiotic introduction, and the abundance of the probiotic organism (i.e., *B. infantis*) was linked to: (1) diminished abundance of taxa associated with preterm neonatal morbidities (Figure 2); (2) with increased capacity for HMO utilization (Figure 4); (3) with reduced ARG abundance (Figure 5); and, (4) with diminished enteric inflammation (Figure 6).

## Conclusions

Overall, the detailed genomic gut microbiome and fecal inflammatory biomarker observations presented here demonstrate that the preterm gut microbiome composition can be altered by feeding *B. infantis* EVC001. Using multivariate modeling, we found that independent of clinical variables associated with degree of prematurity or hospital care, these changes were uniquely related to feeding *B. infantis* EVC001 and that this measure had a significant and beneficial impact on the enteric inflammatory profile. We conclude that the use of *B. infantis* EVC001 in conjunction with human milk in premature infants provides a meaningful and low-risk approach to alter the gut microbiome composition and increase the abundance of a well-established infant gut symbiont that: (1) increased human milk utilization; (2) diminished enteric inflammation; and (3) decreased the abundance of taxa associated with antibiotic-resistance and poor health outcomes (48). Together with human milk feeding, *B. infantis* EVC001 may help to mitigate microbiome-associated risk of morbidity and mortality in hospitalized infants. Future observational and/or controlled studies examining the impact of *B. infantis* EVC001 on preterm infant health outcomes are warranted.

## Materials and Methods

### Study Design

This study was performed at Kaiser Orange County Anaheim and Irvine Neonatal Intensive Care Units (NICUs) under oversight from the Institutional Review Board Kaiser Permanente Southern California (IRB # 12079). Eligible preterm infants were enrolled during the period of May 2019 to October 2019. Inclusion criteria included premature birth < 39 weeks corrected gestational age (cGA) or <1500 grams. Infants born <32 weeks cGA were fed *B. infantis* EVC001 8 × 10^9^ CFU in MCT oil up until 34 cGA as per the standard of care feeding protocol Kaiser Permanente Southern California. At the time of stool collection, site personnel recorded data about the infants and the stool samples and this information was cross-verified by independent hospital staff by comparing de-identified subject and sample metadata from electronic medical records. Data collected from collection logs and electronic medical records included antibiotic exposure on collection days, stool type (meconium, non-meconium, mixed), subject's gestational age at birth, corrected gestational age at sample collection, subject's intake of probiotics, medication, and subject's current diet (i.e., human milk and/or infant formula). Stool samples tubes and stool collection log entry was tagged with linked barcoded labels by the study personnel. Fecal samples were collected from the diaper by site personnel, transferred into sterile collection tubes, and immediately frozen in a designated freezer (-20°C) inside the NICU. Stool samples were collected weekly and shipped on dry ice to the laboratory where they were aliquoted and stored at −80°C for subsequent analysis. A total of 298 fecal samples from 77 premature infants throughout 26–40 weeks corrected gestational age were collected from Anaheim and Irvine NICUs.

### Bacterial DNA Methods

Sufficient DNA was extracted from 296 stool sample aliquots stored in DNA/RNA shield lysis tubes (Zymo Research, Irvine CA) using the ZymoBIOMICS 96 MagBead DNA kit (Zymo Research). Extracted DNA was quantified using QuantIT dsDNA Assay kit, high sensitivity (Thermo Fisher Scientific, Waltham, MA) according to the manufacturer's protocol. Three (3) samples were omitted from downstream analysis due to failure to meet input requirements for library preparation. Libraries were prepared for each sample using the Illumina Nextera DNA Flex library kit (Illumina, San Diego, CA) with unique dual indexes according to manufacturer guidelines. Libraries were pooled and submitted to UC Davis DNA Technologies Core for sequencing on an Illumina NovaSeq S4 flow cell (Illumina, San Diego, CA). Each lane of the S4 flow cell contained 96 libraries.

### Absolute Quantification of *B. infantis* by Quantitative Real-Time PCR

Quantification of the total *B. infantis* was performed by quantitative real-time PCR using Blon_2348 sialidase gene primers Inf2348F (5′-ATA CAG CAG AAC CTT GGC CT-3′), Inf2348_R (5′-GCG ATC ACA TGG ACG AGA AC-3′), and Inf2348_P (5′-/56-FAM/TTT CAC GGA /ZEN/TCA CCG GAC CAT ACG /3lABkFQ/-3′) (64). Each reaction contained 10 μL of 2 × TaqMan Universal Master Mix II with UNG master mix (Thermo Fisher Scientific, Waltham, MA), 0.9 μM of each primer, 0.25 μM probe, and 5 μL of template DNA. Thermal cycling was performed on a QuantStudio 3 Real-Time PCR System and consisted of an initial UNG activation step of 2 min at 50°C followed by a 10-min denaturation at 95°C, succeeded by 40 cycles of 15 s at 95°C and 1 min at 60°C. Standard curves for absolute quantification were generated using genomic DNA extracted from a pure culture of *B. infantis* EVC001.

### Quality Filtering and Removal of Human Sequences

Demultiplexed fastq sequences were quality filtered, including adaptor trimming using Trimmomatic v0.36 (65) with default parameters. Quality-filtered sequences were screened to remove human sequences using GenCoF v1.0 (31) against a nonredundant version of the Genome Reference Consortium Human Build 38, patch release 7 (GRCh38_p7; www.ncbi.nlm.nih.gov).

### Taxonomic and Strain Profiling

Taxonomic profiling of the metagenomic samples was performed using MetaPhlAn2 (66), which uses a library of clade-specific markers to provide pan microbial (bacterial, archaeal, viral, and eukaryotic) profiling (http://hutten-hower.sph.harvard.edu/metaphlan2), in combination with Humann2 (67) to profile functional metagenomics against Uniref90 following the updated global profiling of the Human Microbiome Project (2017) (68). Cross-database annotations (e.g., UniProt to KEGG) were performed within Humman2 using the “utility_mapping” conversion tool package.

### Taxonomic Cluster Grouping Assignment

Species-level taxonomic data consisting of 218 species relative abundance across 292 samples and 77 subjects was used for the analysis. Following the methodology from a previously published study (69), samples were clustered using Jensen–Shannon Divergence (JSD) distance and the Partitioning Around Mediods clustering algorithm. The results were assessed for the optimal number of clusters using the Calinski–Harabasz (CH) Index and Silhouette coefficient. Clustering performed on the full dataset did not show a clear optimal number of clusters. Additionally, uneven sampling between subjects could led to cluster formation dominated by one or a few subjects with a high number of samples. Because of these concerns, subset datasets were created by randomly selecting one sample per subject 1,000 times. Each of the subset datasets were clustered with the same methodology as the full dataset. CH index was computed for iterations from 2 to 6 clusters within each subset. The maximum of those values was chosen as the optimal number of clusters for that subset. Maximum CH index coefficients were stored and observed after the 1,000 cluster iterations and, again, did not show a clear choice for an optimal number of clusters.

Instead, between-class analysis (BCA) was used to identify drivers for clusters. The top *n* important species (where n = twice the number of clusters) were saved for each iteration. Top important taxa were then associated with a specific cluster by examining the angle of importance with the angle of the cluster median on the first two component axes. If the angle was within ±22.5 degrees, that species was considered to be associated with that cluster. Any species shown as both important and associated with a specific cluster at least 800 times over the 1000 iterations was considered a primary driver. Four species (*E. coli*, unclassified *Escherichia* species, *B. longum* species, and *S epidermidis*) met these criteria. Enterotyping of subset datasets proved challenging and inconclusive; therefore, these datasets were further restricted to include only the top cluster-driving bacteria. The CH index and silhouette were examined on the species-restricted datasets, and both metrics pointed to 3 as the optimal number of clusters. BCA was performed again on the species-restricted data and showed a pattern of *B. longum* species, *S. epidermidis*, and *E. coli*/unclassified *Escherichia* clusters. The cluster that each sample fell in over the 1000 iterations was examined. Most samples fell within the same species-driven cluster 100% of the time, and all samples fell within the same cluster at least 90% of the time. Confident that this method of clustering was robust, each sample was then assigned to the cluster it fell into most frequently. The overall diversity between the three observed clusters and the size of the difference between the sample clusters were tested using a permutational multivariate analysis of variance using distance matrices (adonis) obtained from the taxonomic cluster analysis. Alpha diversity was computed at the species level using the Shannon diversity index metric. A linear mixed model was performed in R v.1.1.453 using the package “lme4.” The Shannon diversity indices were used as input to the linear mixed model and compared to several fixed covariates including, whether or not the infants received the probiotic, the age in days postnatal (day of life), and the species cluster assignment, while subject identity was used as a random variable to account for individual variations. *P* values were adjusted using an FDR correction.

### Antibiotic Resistance Gene Analysis

We applied ShortBRED (70, 71) to profile antibiotic resistance (AR) abundance and composition in the infant gut microbiome. We first produced a set of new AR marker sequences by applying ShortBRED-Identify to the Comprehensive Antibiotic Resistance Database (CARD) (72). We then used ShortBRED-Quantify to profile the relative abundance of corresponding antibiotic resistance genes (ARGs). Final values were normalized in Reads Per Kilobase per Million mapped reads (RPKM) to account for sequencing depth as well as gene length. We used custom scripts to collapse CARD individual antibiotic resistance gene entries in their corresponding drug class. Conversion rules are offered within every CARD package update.

### Multiplexed Immunoassays

Interleukin (IL)-1 β, IL-2, IL-4 IL-5, IL-8, IL-10, IL-13, IL-17A, interferon (IFN) γ, and tumor necrosis factor (TNF) α were quantified from 80 mg of frozen stool diluted 1:10 in Meso Scale Discovery (MSD; Rockville, MD) diluent using the U-PLEX Inflammation Panel 1 (human) Kit, and the concentration of fecal calprotectin was quantified using MSD R-PLEX Human Calprotectin Antibody Set. Standards and samples were measured in duplicate, and blank values were subtracted from all readings according to the manufacturer's instructions as previously published (19). All detectable biomarker values were included as continuous data in the analyses; however, values below level of detection (<30% of all cytokines and calprotectin measurements) were generated below the level of quantification to justify parametric statistics. Fecal cytokine and calprotectin concentrations were determined using calibrations curves to which electrochemiluminescence signals were backfitted. Final concentrations were calculated using the Sector Imager 2400 MSD Discovery Workbench analysis software.

### Statistical Analysis

Statistical analyses were performed in R v3.6.2. A Kruskal–Wallis one-way analysis of variance coupled with an FDR or Bonferroni correction was used for statistical comparisons between gene groups, cytokines, and taxa among groups. A *post hoc* Dunn test was applied to determine differences between-species clusters. Statistical analysis to assess total resistome or taxonomic composition by group was performed using a Mann–Whitney or Holm-adjusted Dunn's test. Rarefaction curves were computed to estimate the diversity of the identified ARGs across samples. A nonparametric two-sample *t*-test was used to compare rarefaction curves using Monte Carlo permutations (*n* = 999). To test for the association of microbial abundance with clinical metadata, we performed a multivariate analysis using Multivariate Analysis by Linear Models (MaAsLin2) version for R. Maaslin2 performs boosted, additive general linear models between metadata and microbial abundance (at the taxa level). Boosting of metadata and selection of a model was performed per taxon and, separately, per inflammatory biomarker. The metadata used were total parenteral nutrition (TPN; mL/kg per day), inhaled steroid use associated with sample collection, diaper rash treatment/incidence (e.g., desitin), gestational age at birth (GA, in weeks), formula use associated with sampling, age in days (day of life, DOL), probiotic feeding protocol (EVC001-fed or no probiotic), birth weight (grams), human milk consumption (mL/kg per day), and antibiotic exposure associated with sampling. A linear mixed-effects model was used to examine the effects of probiotic use on ARG acquisition. ARG counts per sample were modeled with probiotic use, day of life, and hospital as fixed effects and hospital and subject as random effects. For random effects, we also examined the degree to which the hospital effect on a given subject deviates from the global effect of hospital. χ^2^ tests were used to compare species cluster compositions between groups, where indicated. Cytokines and *B. infantis*-specific qPCR were correlated with the Spearman method with FDR correction. *P* values throughout the manuscript are represented by asterisks (^****^P < 0.0001; ^***^P < 0.001; ^**^P < 0.01; ^*^P < 0.05).

## Data Availability Statement

Human sequence-filtered raw sequencing reads were deposited in the Sequence Read Archive (SRA; https://www.ncbi.nlm.nih.gov/sra) under the reference number, PRJNA630999.

## Ethics Statement

The study protocol for preterm infants was approved by the Institutional Review Board, Kaiser Permanente Southern California (#12079). Preterm participants were recruited from the neonatal intensive care unit at Kaiser Permanente Southern California, Irvine and Anaheim, California between May 2019 to October 2019. Inclusion criteria included premature birth less than 39 weeks corrected gestational age (cGA) or less than 1500 grams. Parental informed consent was given. Written informed consent from the participants' legal guardian/next of kin was not required to participate in this study in accordance with the national legislation and the institutional requirements.

## Author Contributions

BH, MN, and HH conceived the study. BH, MN, HH, PM, MA, SF, CM, and CH designed the study. SE, MG, C-GO, and CM collected samples analyzed in this study. RM, SC, and GC generated data presented in the manuscript. RM, SK, SC, GC, HB, SF, and BH analyzed the data. MN, GC, SF, and BH wrote the manuscript. All authors approved the final manuscript for publication.

## Conflict of Interest

MN, HH, PM, MA, SE, MG, and C-GO are employees of Southern California Kaiser Permanente, a not for profit healthcare system. C-GO, CH, RM, SK, SC, GC, HB, SF, and BH are employees of Evolve Biosystems, a company focused on restoring the infant microbiome. HH is a member of the Evolve Biosystems Clinical Advisory Board. SF and BH serve as Adjunct Assistant Professors in Food Science & Technology Department, University of Nebraska Lincoln. The remaining author declares that the research was conducted in the absence of any commercial or financial relationships that could be construed as a potential conflict of interest.
