# Supplementary material for: Impact of Probiotic B. infantis EVC001 Feeding in Premature Infants on the Gut Microbiome, Nosocomially Acquired Antibiotic Resistance, and Enteric Inflammation
Source: Front Pediatr. 2021 Feb 16;9:618009. doi: 10.3389/fped.2021.618009 (PMC7921802; doi:10.3389/fped.2021.618009)
Supplement: Supplementary file 1 [file Table_1.PDF]

	shannon entropy		
Predictors	*Estimates*	*CI*	*p*
(Intercept)	1.27	1.03 – 1.51	**<0.001**
evivo_ever2 [yes]	-0.37	-0.63 – -0.10	**0.009**
DOL	0.02	0.01 – 0.02	**<0.001**
Validated_Cluster_group [Escherichia]	0.16	-0.09 – 0.42	0.207
Validated_Cluster_group [Staphylococcus_epidermidis]	-0.29	-0.48 – -0.10	**0.004**
**Random Effects**			
σ^2	0.21		
$\tau_{00\ Subject.ID}$	0.20		
ICC	0.48		
$N_{Subject.ID}$	76		
Observations	292		
Marginal R^2 / Conditional R^2	0.302 / 0.640		
