# Supplementary material for: Impact of Probiotic B. infantis EVC001 Feeding in Premature Infants on the Gut Microbiome, Nosocomially Acquired Antibiotic Resistance, and Enteric Inflammation
Source: Front Pediatr. 2021 Feb 16;9:618009. doi: 10.3389/fped.2021.618009 (PMC7921802; doi:10.3389/fped.2021.618009)
Supplement: Supplementary file 8 [file Data_Sheet_1.PDF]

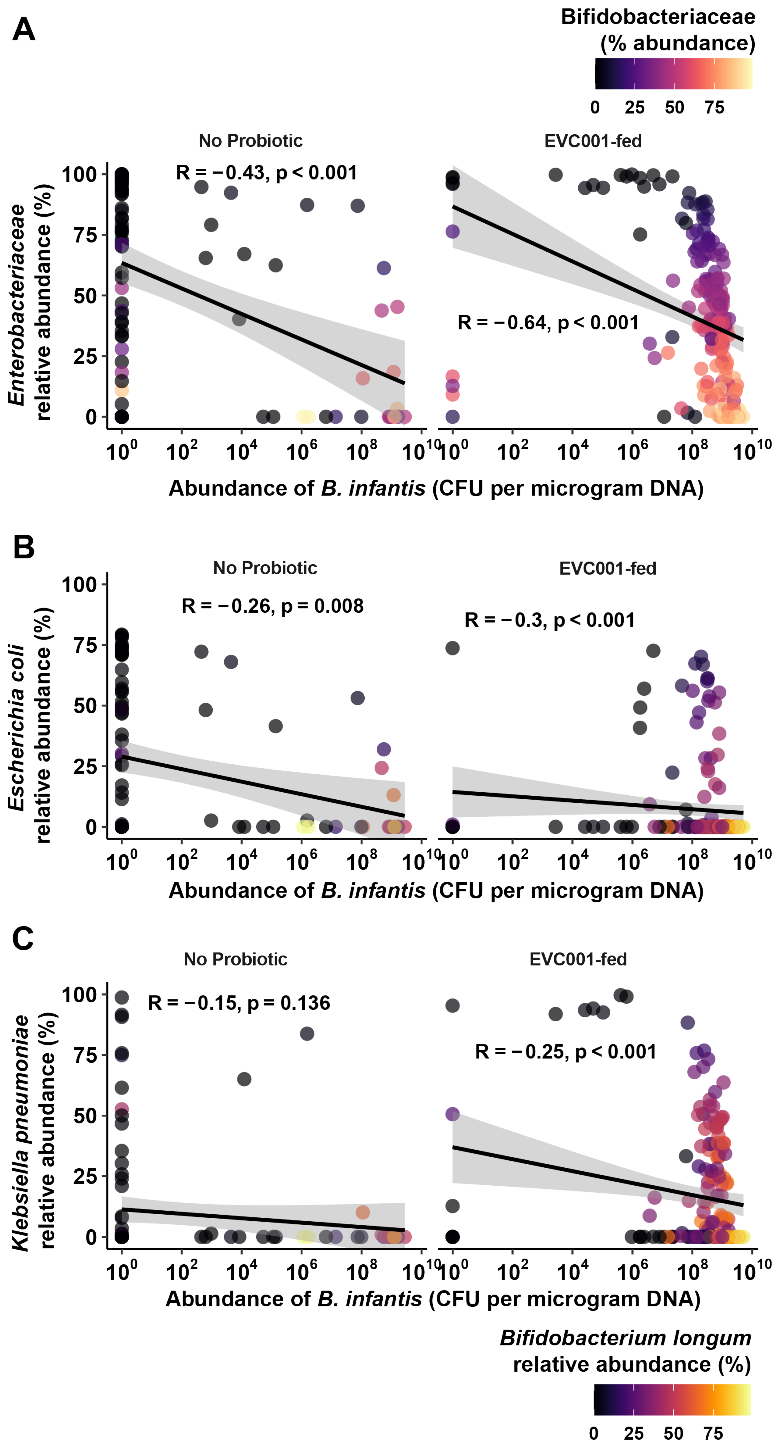

Supplementary Fig 1. ***B. infantis* abundance is negatively correlated with taxa commonly associated with neonatal morbidities.** Confirmation of *B. infantis* abundance, as determined by subspecies-specific qPCR correlations with (A) *Enterobacteriaceae* among samples from infants in each feeding cohort, shaded by the relative abundance of *Bifidobacteriaceae*, (B) Levels of *B. infantis* and the correlation with *Escherichia coli,* shaded by *B. longum* abundance and (C) the abundance of *Klebsiella pneumoniae,* shaded by *B. longum* species abundance. Spearman correlation values are shown, with the linear trend displayed.

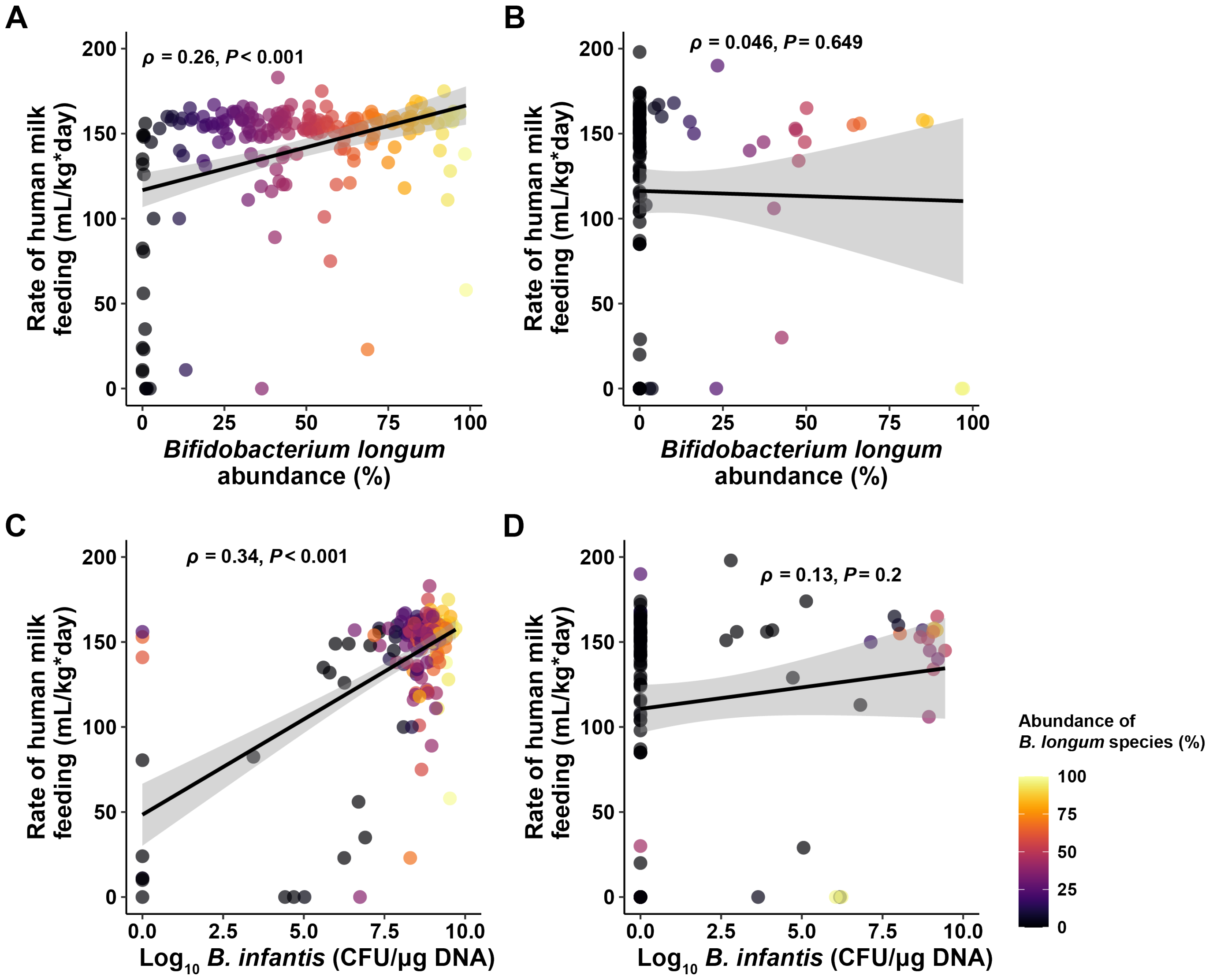

Supplementary Fig 2. ***B. infantis* abundance is associated with human milk feeding.** *Bifidobacterium longum* species abundance (Panel A) is significantly correlated with the rate of human milk feeding (y-axis) among infants fed B. infantis EVC001, but not among infants who were not fed the probiotic (Panel B). This was corroborated by subspecies-specific qPCR for *B. infantis* (Panel C,D) which found the same correlation trends. Points are shaded by the abundance of *B. longum* species in the samples. Spearman correlation values are shown, with the linear trend displayed.

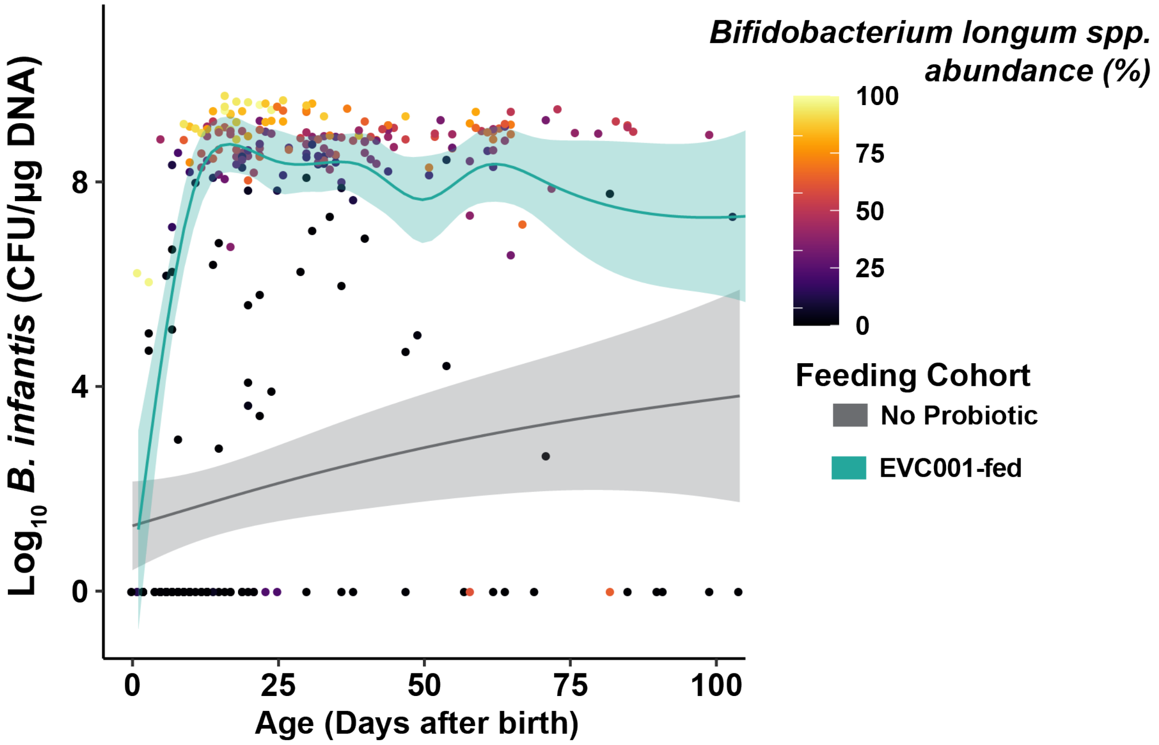

Supplementary Fig 3. ***B. infantis* abundance increases after probiotic feeding.** qPCR using a *B. infantis* subspecies-specific assay demonstrates an increase in the abundance of *B. infantis* among infants fed the probiotic *B infantis* EVC001 (teal trend line and shaded confidence interval), but not among infants who were not (grey trend line and shaded confidence interval). Samples are shaded by the abundance of *B. longum* (relative abundance, %) in each sample, corroborating the increase in *B. longum* species is driven by the abundance of *B. infantis* in the sample.
